# Supplementary material for: MetaQTL: a package of new computational methods for the meta-analysis of QTL mapping experiments
Source: BMC Bioinformatics. 2007 Feb 8;8:49. doi: 10.1186/1471-2105-8-49 (PMC1808479; doi:10.1186/1471-2105-8-49)
Supplement: Additional File 7 — MetaQTL Package : jar file and tutorial. This Zip archive contains both the MetaQTL JAR file and the files of the tutorial. [file 1471-2105-8-49-S7.zip › org.inra.metaqtl/doc/org/metaqtl/bio/IBioAdaptable.html]

IBioAdaptable


|  |  |  |
| --- | --- | --- |
| **Overview** \| **Package** \| **Class** \| **Use** \| **Tree** \| **Deprecated** \| **Index** \| **Help** | |  |
| PREV CLASS   **NEXT CLASS** | **FRAMES**    **NO FRAMES**     **All Classes** |  |
| SUMMARY: NESTED \| FIELD \| CONSTR \| METHOD | DETAIL: FIELD \| CONSTR \| METHOD |  |


---


## org.metaqtl.bio Interface IBioAdaptable

**All Known Subinterfaces:**: IBioAllele, IBioCross, IBioEntity, IBioGenome, IBioIndividual, IBioLGroup, IBioLocus, IBioOntology, IBioOntologyTerm, IBioPopulation

**All Known Implementing Classes:**: AlleleContainer, BioEntity, BioEntityCollection, BioEntityContainer, CrossPopulation, GeneticMap, GroupContainer, Individual, IndividualContainer, LGroup, Locus, LocusContainer, Marker, Ontology, OntologyTerm, Population, QTL

---

``` public interface IBioAdaptable ```

---

| **Method Summary** | |
| --- | --- |
| `IBioAdapter` | `getBioAdapter()` |

**Method Detail**

### getBioAdapter

```
IBioAdapter getBioAdapter()
```


---


|  |  |  |
| --- | --- | --- |
| **Overview** \| **Package** \| **Class** \| **Use** \| **Tree** \| **Deprecated** \| **Index** \| **Help** | |  |
| PREV CLASS   **NEXT CLASS** | **FRAMES**    **NO FRAMES**     **All Classes** |  |
| SUMMARY: NESTED \| FIELD \| CONSTR \| METHOD | DETAIL: FIELD \| CONSTR \| METHOD |  |


---
